# Epidemiology of paediatric inherited arrhythmogenic diseases under “Real World” conditions: findings from a 10-year longitudinal study in Eastern Austria

**DOI:** 10.1007/s00431-025-06646-z

**Published:** 2025-12-03

**Authors:** Sulaima Albinni, Julian Heno, Marianne Gwechenberger, Ina Michel-Behnke, Matthias Gass, Manfred Marx

**Affiliations:** 1 Department of Paediatrics and Adolescent Medicine, Paediatric Heart Centre, Medical University of Vienna, Vienna, Austria; 2 Department of Cardiology, AKH-Vienna, Medical University of Vienna, Vienna, Austria; 3 Paediatric Cardiology, Paediatric Heart Centre, University Children’s Hospital, Zurich, Switzerland

**Keywords:** Sudden cardiac death, Long QT screening, Brugada syndrome children, Arrhythmogenic right ventricular cardiomyopathy, Catecholaminergic polymorphic ventricular tachycardia, IADs, Intergenerational units

## Abstract

Inherited arrhythmogenic diseases (IADs), such as long QT syndrome (LQTS), Brugada syndrome (BrS), arrhythmogenic right ventricular cardiomyopathy (ARVC), catecholaminergic polymorphic ventricular tachycardia (CPVT) and laminopathies (LP), are rare conditions in which arrhythmogenic sudden cardiac death (SCD) may occur as the first symptom. We analysed paediatric prevalence data obtained under “Real World” conditions, defined as a diagnosis based on clinical symptoms, family history or incidentally written ECGs without screening or registry programmes. Findings were compared to published data. This single centre cross-sectional study was performed over a 10-year period in Eastern Austria. We collected all patients with genetically and/or clinically diagnosed IADs (LQTS, SQT, BrS, CPVT, ARVC and laminopathies) and determined the prevalence and paths to diagnosis for each IAD. At the most recent reference date (2024/12/31), 112 patients were enrolled, corresponding to a paediatric prevalence of 1:7000 of all IADs. Subgroup analysis revealed a prevalence of 1:13,000 for LQTS, 1:22,000 for BrS, 1:43,000 for ARVC and 1:368,000 for CVPT. Most patients with LQTS (62%) were diagnosed after incidental ECG, whereas BrS (79%) and ARVC (64%) were diagnosed mainly by family screening. *Conclusion*: This first study on the prevalence of paediatric IADs obtained under “Real World” conditions at a tertiary care centre, reports significantly lower numbers compared to published data. Our findings on patients’ paths to diagnosis suggest that LQTS patients may benefit from selected age-dependent screening programmes, whereas for BrS and ARVC, family screening and intensive collaboration with adult care providers are crucial for diagnosis.

**Table:** 

**What is Known:** • *Although hereditary primary electrical disorders are rare diseases, they may account for 30% or more of all sudden cardiac deaths in the young, making early detection in the general paediatric population crucial.*
**What is New:** • *This is the first study on population-based paediatric prevalence of primary electrical disorders using “Real World” data.* • *The significant lower numbers demand a change in patient acquisition including screening programmes, registries and intensive intergenerational care of families by close cooperation between paediatric and adult cardiologists.*

**Supplementary Information:**

The online version contains supplementary material available at 10.1007/s00431-025-06646-z.

## Introduction

Primary electrical disorders, also called inherited arrhythmogenic diseases (IADs), include disorders without structural abnormalities, also known as cardiac channelopathies, and diseases with structural heart abnormalities, e.g. arrhythmogenic ventricular cardiomyopathy (ARVC) or laminopathies (LP). These disorders may account for up to 30% of all sudden cardiac deaths (SCD) in young individuals (1.3–1.7/100,000 person-years), with twice as many cases in young men and boys than in young women and girls [1–3]. As most studies focus primarily on SCD in athletes, infants and children are vulnerable to be overlooked for potentially lethal cardiovascular diseases [4].

The most common IADs include long QT syndrome (LQTS), Brugada syndrome (BrS) and catecholaminergic polymorphic ventricular tachycardia (CPVT), whereas short QT syndrome is extremely rare. ARVC was present in 10.4% of a series of 200 autopsies of unexpected sudden death cases in adults and children [5]. Laminopathies of the heart, which are very rare genetic conditions, may also lead to sudden arrhythmogenic death and heart failure. All the abovementioned disorders are caused mainly by mutations in genes encoding cardiac ion channels and/or their regulatory proteins, most likely affecting not only one index patient but also various family members within one or more generations. Available paediatric prevalence data on the abovementioned diseases are mainly attributed to screening programmes or are estimated from adult data. Prevalence data of IADs under “Real World” conditions rely on diagnosis based on clinical symptoms, family history or suspicion after incidentally written ECGs. Although this way of identifying patients is common practice, such prevalence data are lacking.

We hypothesised that there might be a significant difference between “Real World” prevalence and published data.

## Methods

This longitudinal observational study was conducted between 2014/1/1 and 2024/12/31. The cross-sectional data were collected at three different time points (2014, 2021, and 2024) by reviewing the patients’ records.

### Inclusion criteria

We included all probands aged 0 to 18 years with genetically and/or clinically diagnosed IADs (LQTS, SQT, BrS, CPVT, ARVC and laminopathies) in Eastern Austria (W, NÖ, Bgl) for whom sufficient information on phenotypic and genotypic presentation, path to diagnosis, cardiac medical history and family history was available. In order to confirm that all patients with IADs in Eastern Austria are referred to our paediatric inherited arrhythmia syndrome unit (IASU), we additionally performed a survey among affiliated paediatric cardiologists to evaluate whether any IAD patients were under external paediatric cardiologists’ care without involvement of our IASU.

### Study design

**Fig. 1:**
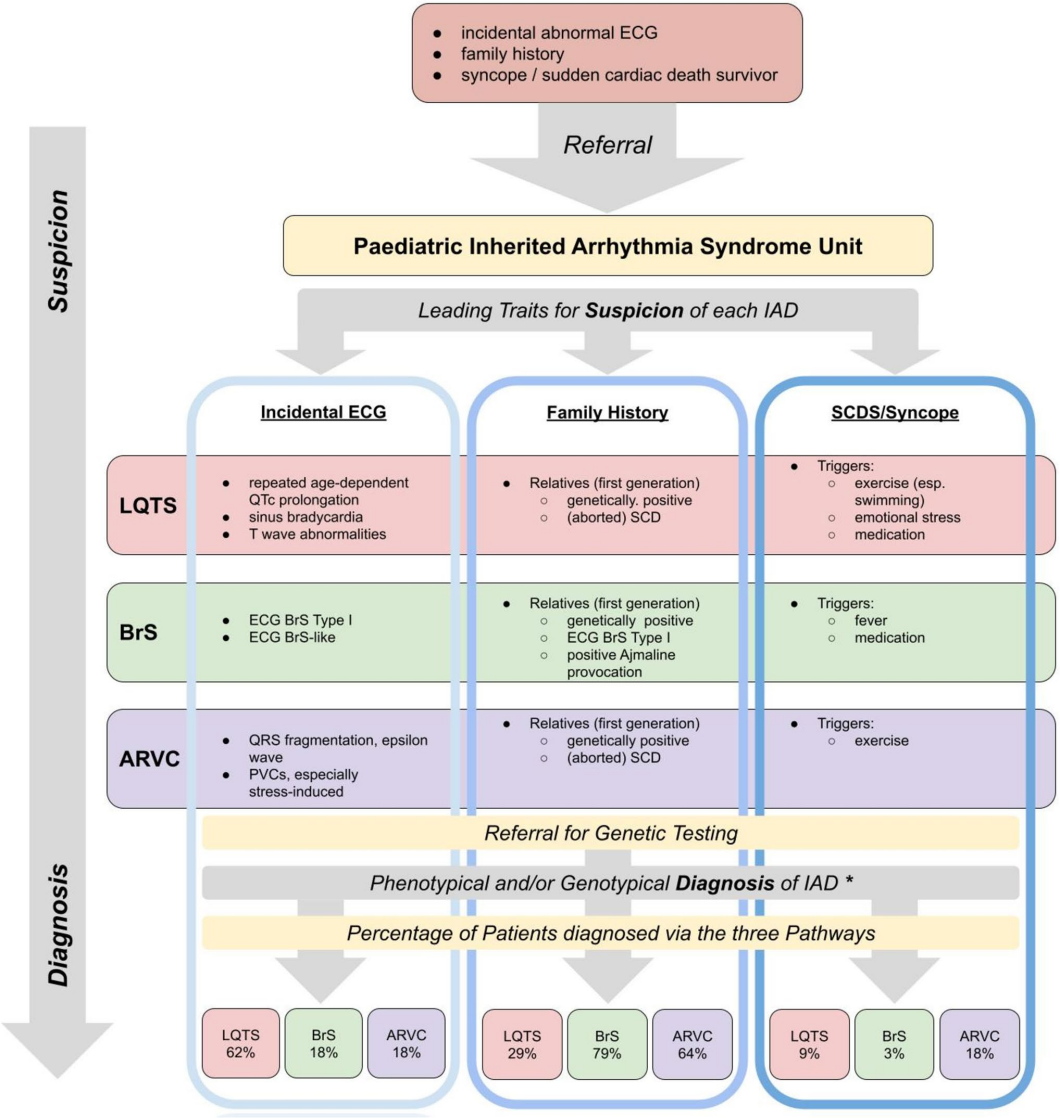
Patients’ paths to diagnosis under “Real World” conditions: Probands were referred to our IASU due to an abnormal incidentally written ECG, positive family history, or a major familial event. In case of persisting suspicion of an IAD after evaluation at our IASU (blue boxes), genetic testing was initiated. *Phenotypical and/or genotypical diagnoses were confirmed according to published consensus (cf. Methods). The distribution of patients diagnosed by each pathway is given at the bottom of the flowchart. Whereas the majority of LQTS patients was diagnosed after an incidentally written ECG (62%) ARVC and BrS were diagnosed mostly due to positive family history (72%, 67%, respectively). IAD, inherited arrhythmogenic disease; IASU, inherited arrhythmia syndrome unit; LQTS, long QT syndrome; ARVC, arrhythmogenic ventricular cardiomyopathy; BrS, Brugada syndrome; SCD, sudden cardiac death

The term “Real World” prevalence assessed in this study stands in contrast to well-known dedicated prospective screening programmes and depends on close cooperation with primary caregivers and in-hospital paediatric cardiologists who refer patients to our IASU in the case of incidentally discovered pathological ECGs, conducted either routinely (e.g. preoperatively, permission for sports, assessment of palpitations or heart murmurs), after clinical indication (unexplained syncope or a BRUE (brief resolved unexplained event)/ALTE (apparent life-threatening event)) or in the context of a positive family history of aborted cardiac arrest (ACA) or SCD. The patients’ path from suspicion of diagnosis to enrolment is illustrated in Fig. 1.

Clinical characteristics, including age at diagnosis, sex, previous cardiac events such as syncope, ventricular fibrillation (VF), ACA or family history of IAD (and/or ACA or SCD), were obtained from the electronic patient data records and are listed in Supplementary Table 1.

When there was a suspicion of LQTS, the QT and RR intervals were manually measured in lead II or V5, and the QT interval was corrected for heart rate (QTc) according to Bazett’s formula.

LQTS was diagnosed as proposed by Priori et al. [6]. In brief, LQTS is diagnosed in the presence of an LQTS risk score ≥ 3.5 in the absence of a secondary cause for QT prolongation and/or in the presence of an unequivocally pathogenic mutation in one of the LQTS genes or in the presence of a QT interval corrected for heart rate using Bazett’s formula (QTc) ≥ 500 ms in a 12-lead electrocardiogram (ECG) and in the absence of a secondary cause for QT prolongation. LQTS can be diagnosed in the presence of a QTc between 480 and 499 ms in repeated 12-lead ECGs in a patient with unexplained syncope in the absence of a secondary cause for QT prolongation and in the absence of a pathogenic mutation.

ECG-based decisions for LQTS genetic testing included QTc prolongation of more than 480 ms or repetitive ECGs with a QTc between 460 and 480 ms. In the presence of pathogenic or likely pathogenic (P/LP) variants in LQTS-associated genes, LQTS was diagnosed regardless of the QT interval. Genetic testing was offered to the probands and their families according to the HRS/EHRA Expert Consensus Statement of Genetic Testing for Channelopathies [7]. Only causative gene mutations classified as definitive or, under certain circumstances, likely pathogenic were used for diagnosis [8, 9].

BrS was diagnosed in probands with typical Brugada type I ECG patterns (spontaneously or after Ajmaline provocation). An ECG was considered to be Brugada type I if the standard 12-lead ECG or a higher right precordial lead ECG (“Brugada ECG position”) fully met the criteria of BrS published in the most recent consensus report [10]. In children, BrS was suspected in cases of typical ECG changes under febrile conditions or in the presence of a “Brugada-like” ECG without underlying structural heart disease (e.g. atrial septal defect). A “Brugada-like” ECG was defined as incomplete or complete RBBB (rsR’ or Rsr’ pattern in lead V1) lacking a distinct isoelectric ST segment to differentiate between depolarization and repolarization. ST-segment elevation was defined as an elevation of the J point of ≥ 0.1 mV in leads V1 through V3.

As BrS is diagnosed mainly in the third or fourth decade of life, most children are identified through family cascade screening [11]. Children with a first degree relative diagnosed with BrS were screened and managed according to the recommendations of the Dutch expert consensus [12]. SCN5A and AKAP9 gene mutations were considered causative for BrS [13].

ARVC was diagnosed using standard diagnostic criteria [14, 15]. Mutations in the genes PKP2, DSP, DSG2, DSC2, JUP and TMEM43 were classified as definitive for ARVC diagnosis [16].

The clinical manifestation of CPVT usually occurs in the first decade of life and is prompted by physical activity or emotional stress [17]. In these cases, genetic examination was initiated [18].

### Genetic testing

All genetic tests were performed by the same certified genetic laboratory (Institute of Medical Genetics, MUW) using a Twist Comprehensive Exome panel covering more than 99% of the genes related to inherited arrhythmias.

### Exclusion criteria

Probands not fulfilling the abovementioned criteria of each IAD as described in detail above were excluded. Patients with premature ventricular complexes (PVCs) or ventricular tachycardia (VT) without signs of IAD were also excluded, as were all probands older than 19 years of age. Probands with mutations classified as variants of unclear significance (VUS) were excluded from the prevalence calculation; however, they are listed in Supplementary Table 1.

### Statistical analysis

Population-based epidemiological data were obtained with an electronic data gathering, analysis and retrieval tool provided by Statistik Austria®^.^ (https://statcube.at/statistik.at/ext/statcube/jsf/tableView/tableView.xhtml) [14]. Statistical analyses were performed using IBM® SPSS® version 29 (Chicago, USA). Datasets were evaluated for normalcy using the Kolmogorov‒Smirnov test, and statistics were accordingly reported using median or mean values. For nonparametric variables, the Kruskal‒Wallis or Mann‒Whitney *U* tests were carried out, and continuous parameters were analysed by Student’s *t* tests. For count analyses, chi-square tests and negative binomial regression analyses were used. Correlations were evaluated using Spearman’s and Pearson’s correlation coefficients, as applicable. Unless otherwise indicated, significance levels are given as two-sided *p* values and considered statistically significant if *p* < 0.05.

## Results

In our catchment area of the eastern federal states (Vienna, Lower Austria and Burgenland), 4.1 million inhabitants, including 740,000 children aged between 0 and 18 years, are registered. Owing to local reasons, we can assume that all the children with suspected IAD were referred to our IASU and seen by us at least once a year. A total of 14/16 affiliated paediatric cardiologists responded to our survey and confirmed full referral adherence.

### Prevalence

Our cross-sectional analysis revealed a constant increase in the number of patients with diagnosed IADs with 37 at the time the unit was set up, tripling from 87 in 2014 to 112 patients at the most recent reference date (2024/12/31). Count comparisons in relation to population data revealed no statistically significant changes.

Within our catchment area comprising 736,864 persons under 18 years of age, our patient numbers corresponded to a population-based prevalence of 1:7000 of all IADs. Subgroup analysis revealed 55 patients with LQTS (24 males/31 females), 33 with BrS (20 males/13 females), 17 with ARVC (9 males/8 females), two with CPVT (2 males) and one with a Lamin A/C mutation (1 female), with a prevalence under “Real World “ conditions of 1:13,000 for LQTS (including VUS 1:12,000), 1:22,000 for BrS (including VUS 1:21,000), 1:43,000 for ARVC (including VUS 1:40,000) and 1:368,000 for CVPT (including VUS 1:246,000) (Table 1).

**Table 1:** Overview of paediatric IADs prevalence (rounded to the nearest 1 000; VUS excluded) for East Austria. Population numbers according to *Statistik Austria*. (IADs Inherited arrhythmogenic diseases, LQTS Long QT syndrome, BrS Brugada syndrome, ARVC arrhythmogenic right ventricular cardiomyopathy, CPVT catecholaminergic polymorphic ventricular tachycardia, Lamin A/C and Laminopathy A/C, Others: 2 × Short QTS, 1 × V-Fib, 1 × Sinus node disease)

	Eastern Austria in total				
Reference date	2014 2014/12/31	2021 Reference date 2021/12/31		2024 Reference date 2024/12/31	
Population 0–18y	674,555	714,826		736,864	
	*n*	*n*	Prevalence (rounded)	*n*	Prevalence (rounded)
LQTS	20	45	1:16,000	55	1:13,000
LQTS 1	10	24	1:30,000	27	1:27,000
LQTS 2	4	13	1:5000	16	1:46,000
LQTS 3	3	4	1:179,000	1	
LQTS 4		1		1	
LQTS 7		2	1:357,000	3	1:246,000
LQTS 8				4	1:184,000
LQTS phenotypical positive, genetic neg		1		3	1:246,000
BrS	5	24	1:30,000	33	1:22,000
ARVC	4	11	1:65,000	17	1:43,000
CPVT	3	4	1:179,000	2	1:368,000
Lamin A/C	5	3	1:238,000	1	
Others				4	1:184,000
Total	37	87	1:8 000	112	1:7000

Among the included patients, four were categorised as “others”, of whom two had SQTS, one had idiopathic V-Fib and one had sinus node disease. Chi-square testing revealed no change in the frequency of subdiagnoses (LQTS, BrS, ARVC) in relation to the entire proband cohort between 2014 and 2024.

**Fig. 2:**
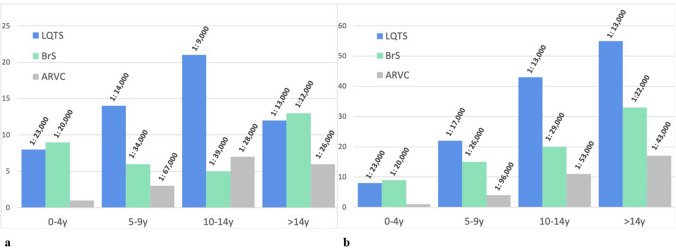
**a** Bar charts showing the number of probands (as of 2024) with the most common IADs in our care subdivided into age groups, with calculated prevalence using population data of the indicated age group, respectively (oblique). **b** Same data presented as cumulative age group prevalence

The median age was 12 years (1–18 years) for all patients, 12 years (2–18 years) in the LQTS group, 10 years (3–18 years) in the BrS group, 13 years (1–18 years) in the ARVC group and 13 years (12–16 years) in the CPVT group. The patient with L A/C mutation was 18 years of age at the time of diagnosis. Age distributions, prevalence according to age groups and cumulative prevalence within the various groups are illustrated in Fig. 2. Statistically, the age at the time of first referral to our IASU did not differ significantly among the diagnoses, nor did the sex distribution within each group.

In comparison to available prevalence data published in the literature, our prevalence data obtained under “Real World” conditions were significantly lower (Table 2).

**Table 2:** “Real World” IADs—prevalence in Eastern Austria in comparison to the literature (reference date 2024/12/31, rounded to the nearest 1 000). Note the different ages at clinical disease expression and that—besides LQTS—data are compared with analysis mainly derived from adult studies [15, 23, 24, 29, 30]

**Population** *n* = 736 864	**“Real World” numbers**	**“Real World” prevalence** (rounded)	**Literature prevalence**	**Expected numbers** according to the literature
LQTS 1–13	55	1:13,000	1:2000	368
BrS	33	1:22,000	1:5000–2000	147–368
ARVC	17	1:43,000	1:5000–2000	147–368
CPVT	2	1:368,000	1:10,000	74
Lamin A/C	1	1:737,000	Extremely rare	?

### Genetics

Genetic testing was performed in 106/112 probands (3 refused and 3 with a negative genetic result for an index patient). Causative mutations were found in 93/106 probands, corresponding to an overall diagnostic yield of 88%.

In LQTS, positive genetic testing was reported for 51/55 probands, with a diagnostic yield of 94% (27 with KCNQ1, 16 with KCNH2, 1 with SCN5A, 2 with KCNJ2, 3 with CACNAC1, 1 with Ankyrin, 1 with CACNA1E, 3 negative and 1 pending).

Among the 33 BrS patients, a causative genetic mutation was detected in 20/27 probands (18 with SCN5A, 2 with AKAP9, 6 negative, 1 pending, 3 refused, 3 not performed because of a negative result for the index patient), equalling a diagnostic yield of 77%. Among the 6 genetically negative children, 3 had a parent with a positive Ajmaline test and/or an ICD implanted as secondary prevention. The remaining three patients had a BrS-like ECG.

The diagnostic yield of ARVC was 89%, with causative mutations in 15/17 probands (4 with DSP, 4 with PKP2, 4 with DSG2, 1 with RYR2, 1 with TMEM43, 1 with CACNa1AC, and 2 were negative). Both patients with CPVT had a CASQ2 mutation. One patient had a Lamin A/C mutation.

Among the remaining four patients categorized as “others”, two patients with SQTS were genetically negative, one with symptomatic SA block had a KCNJ5 mutation, and one with V-Fib had a SCN5A mutation.

### Path to diagnosis

Diagnostic pathways differed significantly among the various IAD groups.

A total of 34/55 patients with LQTS were diagnosed because of QTc prolongation on incidentally obtained ECGs. Overall, the mean QT interval was 493 ms ± 25.6 (median 484 ms; IQR 471–511 ms). For LQT1, the mean interval was 496 ms ± 21.5 (median 503 ms; IQR 480–513 ms), and for LQT2, the mean interval was 481 ms ± 15.2 (median 480 ms; IQR 467–493 ms).

A total of 16/55 patients were diagnosed by family cascade screening after either diagnosis (10/16) or ACA/SCD (6/16) in a family member. The remaining five patients were diagnosed after syncopal events during exercise.

The diagnosis among the 33 patients with BrS was mainly established in the context of family screening (26/33), either because of a typical Brugada ECG pattern or because of ACA/SCD in an adult family member. Six patients were diagnosed after presenting with a “Brugada-like ECG” (*n* = 4) or a BrS type I ECG under febrile conditions (*n* = 2). One patient experienced syncope during fever. Notably, compared with female probands, male probands with BrS were more likely to have been diagnosed after an event or because of a suspicious incidental ECG, who were most likely to be diagnosed as part of family screening (*p* = 0.038).

In the ARVC group, 11/17 probands were referred because of a positive family history of either cardiomyopathy (*n* = 2), SCD (*n* = 4) or ACA (4) among family members. Of those, 3 had ECG abnormalities, such as PVC or VTs, at presentation. Three patients were diagnosed after presenting with PVCs and/or syncope during exercise and three were diagnosed via an incidental ECG.

In all patients with CPVT, a diagnosis was suspected after syncope during exertion, where a Holter ECG typically documented increasing PVCs and polymorphic VTs with increasing heart rate. The patient with Lamin A/C mutation was referred because of familial SCD and had a low-voltage ECG.

Clinical characteristics, including age at diagnosis, IAD subgroup, genetics and path to diagnosis, are listed in Supplementary Table 1.

## Discussion

With respect to East Austria, which has approximately four million inhabitants, our IASU is the only dedicated institution that has reviewed a total of approximately 150 paediatric patients over the past decade.

Owing to a continuous transition to adult care, numbers are always fluctuating, explaining our actual count of 112 patients (125 including patients with VUS) at the last cross-sectional reference date. However, despite these fluctuations, there has been an overall constant increase in patient numbers over the past 10 years, which may be in part due to a 15% increase in the population. Moreover, this is founded on the burden of responsibility for patients with potentially life-threatening diseases and—at least in the beginning—the lack of genetic analysis capacity outside a tertiary care centre, both leading to a trustful cooperation with other hospitals and practitioners in East Austria and thereby ensuring data consistency over the past decade. Notably, our survey, performed among paediatric cardiologists, confirmed consistent referral adherence.

Despite these efforts, the difference between our “Real World” prevalence and the expected numbers according to the published prevalence data is still significant. However, our prevalence depends on a suspicion of diagnosis due to clinical reasons or family history, which is a completely different approach from the available data obtained from screening studies. Furthermore, molecular autopsies are not performed routinely after sudden death, particularly in children. Addressing this issue in our country would not only influence prevalence data but also improve preventive personalized measures to reduce the risk of malignant arrhythmias and sudden death among the relatives of victims.

### LQTS

For LQTS, the paediatric prevalence is based on the findings of a community-based screening program performed by Schwartz et al. 15 years ago [19]. In this pivotal study, the authors revealed a prevalence close to 1:2000 for phenotypically and/or genetically proven LQTS, which remains the highest published prevalence in the Caucasian population thus far. The same group analysed the cost-effectiveness of nationwide screening programmes and reported that neonatal ECG screening was feasible and cost-effective for preventing unnecessary deaths during infancy and childhood [20]. Furthermore, the probability of a diagnosis of LQTS in a school-based screening program performed in Japan was found to be 1:3300 for subjects aged 6 years and 1:1000 for those aged 12 years [21]. However, according to a study published in 2010, most paediatric cardiologists are still sceptical of ECG screening for LQTS [22], and screening programmes are not established in most countries. Our findings obtained under “Real World” conditions describe the opposite approach to diagnosing these conditions. However, our prevalence of 1:13,000 is almost sevenfold lower than that published by Schwartz et al. although we used the same inclusion criteria (genetic testing if repetitive ECGs with QTc between 460 and 480 ms or higher) and can ensure extensive referral adherence. One of the possible reasons might be the age-dependent detection rate caused by the lower frequencies of ECGs conducted in infancy and early childhood, with the consequence that some affected infants and younger children escape early diagnosis by “Real World” approach.

A recently published study of cardiovascular preparticipation screening (PPS) in 22,000 consecutive young competitive athletes revealed that compared with a single-only PPS, a repeat cardiovascular evaluation during an 11-year study period increased the diagnostic yield of cardiovascular disease at risk of SCD and that the peak diagnostic yield is among children older than 12 years [23]. This finding is in accordance with our findings, revealing a higher frequency of LQTS detection in older children, most likely due to an increasing number of recorded ECGs with increasing age (Fig. 2).

Finally, given the variation in the prevalence of LQTS resulting from different diagnostic approaches for a disease with a possibly high burden even in childhood, it seems reasonable to rethink the value of LQTS screening or staged screening programmes in adolescence to detect asymptomatic probands.

Genetic testing at our institution was found to have a relatively high diagnostic yield of 94%, which might be explained by the strict selection of patients. Ideal screening should produce as few false-positive results as possible while detecting probands who are at risk. Given that the threshold QTc interval used for diagnosis is the primary determinant of test sensitivity and specificity, a false-positive rate for those with a QTc interval of > 460 ms is less than 1 in 1000 but 4 in 1000 for those with a QTc interval of > 450 ms [4]. At our institution, genetic testing is initiated in the case of repeated QTc intervals > 460 ms, which certainly contributes to the high diagnostic yield. Notably, we regularly measure the QT interval manually and calculate the QTc with Bazett’s equation to take sinus arrhythmia into account and thereby avoid false-positive results based on automatically derived QTc intervals, which may contrast with other institutions with lower diagnostic yields of genetic testing (30% to 70%) [24, 25].

Furthermore, genetic testing revealed eight carriers of VUS, which is a challenge in clinical practice, most likely raising more questions than answers. Interestingly, most of them presented significant phenotypic expression, with QTC intervals ≥ 500 ms (Supplementary Table 1). Despite the unclear significance of the mutations, we consider these patients at risk and follow them like genetically positive patients, given that some variants might be reclassified in the future to be pathogenic.

### BrS

BrS is described as a Mendelian syndrome with an autosomal dominant inheritance pattern and incomplete penetrance [26]. Different studies propose varying adult prevalences ranging from 1:5000 to 1:2000 [27, 28], depending on region and ethnicity. Its prevalence is higher in Asia and the Middle East, where estimates range between 1:270 and 1:625 [29]. The current literature on paediatric prevalence is restricted to Japanese school-based screening programmes, suggesting much lower numbers in children despite growing evidence of disease onset early in childhood [30]. As BrS is clinically and phenotypically expressed mainly in the third or fourth decade of life, most children are identified by family cascade screening [11]. As such, patients enrolled in our study were diagnosed either in the context of extended family screening or, less commonly, after presenting with a Brugada-like ECG. Our prevalence of 1:22,000 obtained under “Real World” conditions is four to ten times lower than that in adults and may result from incomplete referrals of family members after diagnosis of an adult index patient. In recent years, we have offered genetic analyses to all family members. While a SCN5A mutation can be found in only 20–25% of the adult population with BrS [31], the prevalence of a gene mutation in paediatric patients is higher, reaching 58.1% [32], which is in accordance with our current positive yield of 66%.

#### ARVC

ARVC should be considered in adolescents or young adults who present with symptoms of palpitations, PVCs, syncope, or aborted SCD. The adult prevalence of ARVC is estimated to be between 1:5000 and 1:2000 [33, 34], whereas our paediatric prevalence was 1:43,000, with a median patient age of 13 years (1–18 years). While age-related penetrance is evident in ARVC, with the highest incidence occurring between the ages of 30 and 40 years, it is noteworthy that no comprehensive studies have been conducted to systematically assess ARVC epidemiology during childhood [35].

Although ARVC phenotypically presents as “cardiomyopathy”, resulting in high alertness among adult cardiologists, the diagnosis in our patients was made mainly by a paediatric cardiologist, including adult siblings of children who were referred to us after unexplained SCD of a family member. Our significantly lower prevalence of BrS and ARVC than in adults is attributed to the fact that in common practice, only patients with clinical features or family history can be diagnosed. As shown in our analysis, particularly for BrS and ARVC, family history is the key to diagnosis in children. This highlights the necessity of intensive collaboration with adult cardiologists, including even more generous referrals of paediatric family members, not only in the case of an IAD diagnosis but also after unexplained SCD of an adult family member. In addition, as disease penetrance increases with age and peaks in young adulthood, our numbers might be equally correct. However, given the paucity of paediatric prevalence studies, we sought to compare our results to available adult data to highlight that higher detection rates should be expected, predominantly in adolescence or young adulthood.

#### CPVT

The literature-reported estimates of overall CPVT prevalence are 1:10,000 or less [6, 36, 37]. The population-based paediatric prevalence might be uncertain, as CPVT is difficult to diagnose and easily missed, especially in patients in whom SCD is the first manifestation at a young age.

Our prevalence of 1:368 000 is very low, and the gap in published numbers might illustrate the difficulties of suspecting this diagnosis, as ECG at rest will generally be normal. Presumably, we still need to increase alertness for this disease among primary health care providers and paediatric cardiologists in cases of unclear fainting or syncope associated with stressful situations or activities, as CPVT is associated with an annual near-fatal and fatal event rate of 1.9%, decreasing to 0.8% in treated patients [38].

Limitations of this analysis include the small number of patients and that the study was conducted only in the eastern part of Austria. The design of our study implies that our prevalence is dependent on the alertness and cooperation of referring colleagues in the paediatric and adult fields. Although we can largely confirm referral adherence in our catchment area, we cannot exclude the loss of some patients. However, even if a substantial number of additional patients were treated by other colleagues or hospitals not reached by our survey, our prevalence numbers would still be very low. Comparisons of our “Real World” prevalence with available data should be performed with caution because of the different study designs used.

As a tertiary care centre, we are dependent on referrals. Practitioners and referring hospital colleagues were not referring probands with QTc values below 460 ms without other symptoms, thus, there might be some LQTS patients who did not reach our IAD tertiary unit. Furthermore, probands with a Schwartz score between 1.5 and 3 are very likely to remain undiagnosed, as repolarization abnormalities or bradycardia in an ECG conducted in a general paediatric setting are rarely the reason for referral, particularly in the absence of symptoms or family history. We excluded probands with VUS from the statistical analysis, which additionally decreased our prevalence data.

## Conclusions

This is the first study on prevalence of paediatric IADs obtained under “Real World” conditions during a 10-year longitudinal period. Our findings suggest a significantly lower prevalence than published and merit confirmation by further paediatric multicentre studies. Furthermore, our detailed analysis of patients’ pathways to diagnosis illustrates common diagnostic algorithms and highlights where improvements might still be needed. In particular, LQTS patients may benefit from selected age-dependent prospective screening programmes sharing the concept of repetitive ECGs, whereas for BrS and ARVC, a family history and intensive collaboration with adult care providers are key factors for early diagnosis. Finally, we believe that the intergenerational care of families may be substantially improved by close networking between paediatric and adult cardiologists and primary health care providers linked by dedicated IAD tertiary units.

## Supplementary Information

Below is the link to the electronic supplementary material.

ESM 1 (DOCX 60.7 KB)

## Data Availability

No datasets were generated or analysed during the current study.

## References

[1] Puranik R, Chow CK, Duflou JA et al (2005) Sudden death in the young. Heart Rhythm 2:1277–1282. 10.1016/j.hrthm.2005.09.008 16360077 10.1016/j.hrthm.2005.09.008

[2] Ha FJ, Han H-C, Sanders P et al (2020) Sudden cardiac death in the young: incidence, trends, and risk factors in a nationwide study. Circ Cardiovasc Qual Outcomes 13:e006470. 10.1161/CIRCOUTCOMES.119.006470 33079584 10.1161/CIRCOUTCOMES.119.006470

[3] Couper K, Putt O, Field R, Poole K, Bradlow W, Clarke A, Perkins GD, Royle P, Yeung J, Taylor-Phillips S (2020) Incidence of sudden cardiac death in the young: a systematic review. BMJ Open 10(10):e040815. 10.1136/bmjopen-2020-040815 33033034 10.1136/bmjopen-2020-040815 PMC7542928

[4] Saul JP, Schwartz PJ, Ackerman MJ, Triedman JK (2014) Rationale and objectives for ECG screening in infancy. Heart Rhythm 11:2316–2321. 10.1016/j.hrthm.2014.09.047 25239430 10.1016/j.hrthm.2014.09.047 PMC4254269

[5] Tabib A, Loire R, Chalabreysse L et al (2003) Circumstances of death and gross and microscopic observations in a series of 200 cases of sudden death associated with arrhythmogenic right ventricular cardiomyopathy and/or dysplasia. Circulation 108:3000–3005. 10.1161/01.CIR.0000108396.65446.21 14662701 10.1161/01.CIR.0000108396.65446.21

[6] Priori SG, Wilde AA, Horie M, Cho Y, Behr ER, Berul C, Blom N, Brugada J, Chiang CE, Huikuri H, Kannankeril P, Krahn A, Leenhardt A, Moss A, Schwartz PJ, Shimizu W, Tomaselli G, Tracy C (2013) HRS/EHRA/APHRS expert consensus statement on the diagnosis and management of patients with inherited primary arrhythmia syndromes: document endorsed by HRS, EHRA, and APHRS in May 2013 and by ACCF, AHA, PACES, and AEPC in June 2013. Heart Rhythm 10(12):1932–1963. 10.1016/j.hrthm.2013.05.014 24011539 10.1016/j.hrthm.2013.05.014

[7] Ackerman MJ, Priori SG, Willems S et al (2011) HRS/EHRA expert consensus statement on the state of genetic testing for the channelopathies and cardiomyopathies: this document was developed as a partnership between the Heart Rhythm Society (HRS) and the European Heart Rhythm Association (EHRA). Europace 13:1077–1109. 10.1093/europace/eur245 21810866 10.1093/europace/eur245

[8] Adler A, Novelli V, Amin AS et al (2020) An international, multicentered, evidence-based reappraisal of genes reported to cause congenital long QT syndrome. Circulation 141:418–428. 10.1161/CIRCULATIONAHA.119.043132 31983240 10.1161/CIRCULATIONAHA.119.043132 PMC7017940

[9] Aiba T, Ohno S, Takegami M et al (2025) Clinical impact of genetic testing for long QT syndrome-evidence from a nationwide LQTS registry in Japan. Circ J 89:835–844. 10.1253/circj.CJ-25-0105 40159220 10.1253/circj.CJ-25-0105

[10] Antzelevitch C, Yan G-X, Ackerman MJ et al (2017) J-wave syndromes expert consensus conference report: emerging concepts and gaps in knowledge. Europace 19:665–694. 10.1093/europace/euw235 28431071 10.1093/europace/euw235 PMC5834028

[11] Minier M, Probst V, Berthome P et al (2020) Age at diagnosis of Brugada syndrome: influence on clinical characteristics and risk of arrhythmia. Heart Rhythm 17:743–749. 10.1016/j.hrthm.2019.11.027 31790831 10.1016/j.hrthm.2019.11.027

[12] Peltenburg PJ, Hoedemaekers YM, Clur SaB et al (2023) Screening, diagnosis and follow-up of Brugada syndrome in children: a Dutch expert consensus statement. Neth Heart J 31:133–137. 10.1007/s12471-022-01723-6 36223066 10.1007/s12471-022-01723-6 PMC9554382

[13] Hosseini SM, Kim R, Udupa S et al (2018) Reappraisal of reported genes for sudden arrhythmic death: evidence-based evaluation of gene validity for Brugada syndrome. Circulation 138:1195–1205. 10.1161/CIRCULATIONAHA.118.035070 29959160 10.1161/CIRCULATIONAHA.118.035070 PMC6147087

[14] Marcus FI, McKenna WJ, Sherrill D, Basso C, Bauce B, Bluemke DA, Calkins H, Corrado D, Cox MGPJ, Daubert JP, Fontaine G, Gear K, Hauer R, Nava A, Picard MH, Protonotarios N, Saffitz JE, Sanborn DMY, Steinberg JS, Tandri H, Thiene G, Towbin JA, Tsatsopoulou A, Wichter T, Zareba W (2010) Diagnosis of arrhythmogenic right ventricular cardiomyopathy/dysplasia: proposed modification of the Task Force Criteria. Eur Heart J 31(7):806–814. 10.1093/eurheartj/ehq025 20172912 10.1093/eurheartj/ehq025 PMC2848326

[15] Conte G, Scherr D, Lenarczyk R, Gandjbachkh E, Boulé S, Spartalis MD, Behr ER, Wilde A, Potpara T (2020) Diagnosis, family screening, and treatment of inherited arrhythmogenic diseases in Europe: results of the European Heart Rhythm Association survey. Europace 22(12):1904–1910. 10.1093/europace/euaa223 33367591 10.1093/europace/euaa223

[16] James CA, Jongbloed JDH, Hershberger RE, Morales A, Judge DP, Syrris P, Pilichou K, Domingo AM, Murray B, Cadrin-Tourigny J, Lekanne Deprez R, Celeghin R, Protonotarios A, Asatryan B, Brown E, Jordan E, McGlaughon J, Thaxton C, Kurtz CL, van Tintelen JP (2021) International evidence based reappraisal of genes associated with arrhythmogenic right ventricular cardiomyopathy using the clinical genome resource framework. Circ Genom Precis Med 14(3):e003273. 10.1161/CIRCGEN.120.003273 33831308 10.1161/CIRCGEN.120.003273 PMC8205996

[17] Priori SG, Napolitano C, Memmi M et al (2002) Clinical and molecular characterization of patients with catecholaminergic polymorphic ventricular tachycardia. Circulation 106:69–74. 10.1161/01.cir.0000020013.73106.d8 12093772 10.1161/01.cir.0000020013.73106.d8

[18] Walsh R, Adler A, Amin AS et al (2022) Evaluation of gene validity for CPVT and short QT syndrome in sudden arrhythmic death. Eur Heart J 43:1500–1510. 10.1093/eurheartj/ehab687 34557911 10.1093/eurheartj/ehab687 PMC9009401

[19] Schwartz PJ, Stramba-Badiale M, Crotti L et al (2009) Prevalence of the congenital long-QT syndrome. Circulation 120:1761–1767. 10.1161/CIRCULATIONAHA.109.863209 19841298 10.1161/CIRCULATIONAHA.109.863209 PMC2784143

[20] Quaglini S, Rognoni C, Spazzolini C, Priori SG, Mannarino S, Schwartz PJ (2006) Cost-effectiveness of neonatal ECG screening for the long QT syndrome. Eur Heart J 27(15):1824–1832. 10.1093/eurheartj/ehl115 16840497 10.1093/eurheartj/ehl115

[21] Yoshinaga M, Kucho Y, Nishibatake M et al (2016) Probability of diagnosing long QT syndrome in children and adolescents according to the criteria of the HRS/EHRA/APHRS expert consensus statement. Eur Heart J 37:2490–2497. 10.1093/eurheartj/ehw072 27026747 10.1093/eurheartj/ehw072

[22] Chang R-KR, Rodriguez S, Gurvitz MZ (2010) Electrocardiogram screening of infants for long QT syndrome: survey of pediatric cardiologists in North America. J Electrocardiol 43:4–7. 10.1016/j.jelectrocard.2009.07.004 19665725 10.1016/j.jelectrocard.2009.07.004

[23] Sarto P, Zorzi A, Merlo L et al (2023) Value of screening for the risk of sudden cardiac death in young competitive athletes. Eur Heart J. 10.1093/eurheartj/ehad017 36760222 10.1093/eurheartj/ehad017 PMC10027466

[24] Stava TT, Berge KE, Haugaa KH, Smedsrud MK, Leren TP, Bogsrud MP (2024) Molecular genetics in 1991 arrhythmia probands and 2782 relatives in Norway: results from 17 years of genetic testing in a national laboratory. Clin Genet 106(5):585–602. 10.1111/cge.14593 39073097 10.1111/cge.14593

[25] Hofman N, Tan HL, Alders M et al (2013) Yield of molecular and clinical testing for arrhythmia syndromes: report of 15 years’ experience. Circulation 128:1513–1521. 10.1161/CIRCULATIONAHA.112.000091 23963746 10.1161/CIRCULATIONAHA.112.000091

[26] Brugada P, Brugada J (1992) Right bundle branch block, persistent ST segment elevation and sudden cardiac death: a distinct clinical and electrocardiographic syndrome. A multicentre report. J Am Coll Cardiol 20:1391–1396. 10.1016/0735-1097(92)90253-j 1309182 10.1016/0735-1097(92)90253-j

[27] Chockalingam P, Wilde A (2012) The multifaceted cardiac sodium channel and its clinical implications. Heart 98:1318–1324. 10.1136/heartjnl-2012-301784 22875823 10.1136/heartjnl-2012-301784

[28] Brugada J, Campuzano O, Arbelo E, Sarquella-Brugada G, Brugada R (2018) Present status of Brugada syndrome: JACC state-of-the-art review. J Am Coll Cardiol 72(9):1046–1059. 10.1016/j.jacc.2018.06.037 30139433 10.1016/j.jacc.2018.06.037

[29] Marsman EMJ, Postema PG, Remme CA (2022) Brugada syndrome: update and future perspectives. Heart 108:668–675. 10.1136/heartjnl-2020-318258 34649929 10.1136/heartjnl-2020-318258

[30] Yamakawa Y, Ishikawa T, Uchino K et al (2004) Prevalence of right bundle-branch block and right precordial ST-segment elevation (Brugada-type electrocardiogram) in Japanese children. Circ J 68:275–279. 10.1253/circj.68.275 15056820 10.1253/circj.68.275

[31] Campuzano O, Sarquella-Brugada G, Cesar S et al (2020) Update on genetic basis of Brugada syndrome: monogenic, polygenic or oligogenic? Int J Mol Sci 21:7155. 10.3390/ijms21197155 32998306 10.3390/ijms21197155 PMC7582739

[32] Michowitz Y, Milman A, Andorin A, Sarquella-Brugada G, Gonzalez Corcia MC, Gourraud J-B, Conte G, Sacher F, Juang JJM, Kim S-H, Leshem E, Mabo P, Postema PG, Hochstadt A, Wijeyeratne YD, Denjoy I, Giustetto C, Mizusawa Y, Huang Z, Jespersen CH, Maeda S, Takahashi Y, Kamakura T, Aiba T, Arbelo E, Mazzanti A, Allocca G, Brugada R, Casado-Arroyo R, Champagne J, Priori SG, Veltmann C, Delise P, Corrado D, Brugada J, Kusano KF, Hirao K, Calo L, Takagi M, Tfelt-Hansen J, Yan G-X, Gaita F, Leenhardt A, Behr ER, Wilde AAM, Nam G-B, Brugada P, Probst V, Belhassen B (2019) Characterization and management of arrhythmic events in young patients with Brugada syndrome. J Am Coll Cardiol 73(14):1756–1765. 10.1016/j.jacc.2019.01.048 30975291 10.1016/j.jacc.2019.01.048

[33] Monasky MM, Micaglio E, Ciconte G, Pappone C (2020) Brugada syndrome: oligogenic or Mendelian disease? Int J Mol Sci. 10.3390/ijms21051687 32121523 10.3390/ijms21051687 PMC7084676

[34] Dalal D, Nasir K, Bomma C et al (2005) Arrhythmogenic right ventricular dysplasia: a United States experience. Circulation 112:3823–3832. 10.1161/CIRCULATIONAHA.105.542266 16344387 10.1161/CIRCULATIONAHA.105.542266

[35] Mariani MV, Pierucci N, Fanisio F, Laviola D, Silvetti G, Piro A, La Fazia VM, Chimenti C, Rebecchi M, Drago F, Miraldi F, Natale A, Vizza CD, Lavalle C (2024) Inherited arrhythmias in the pediatric population: an updated overview. Medicina Kaunas 60(1):94. 10.3390/medicina60010094 38256355 10.3390/medicina60010094 PMC10819657

[36] Napolitano C, Priori SG, Bloise R (1993) Catecholaminergic polymorphic ventricular tachycardia. In: Adam MP, Ardinger HH, Pagon RA, et al (eds) GeneReviews®. University of Washington, Seattle, Seattle (WA) 20301466

[37] Bratincsák A, Kimata C, Limm-Chan BN, Vincent KP, Williams MR, Perry JC (2020) Electrocardiogram standards for children and young adults using Z-scores. Circ Arrhythm Electrophysiol 13(8):e008253. 10.1161/CIRCEP.119.008253 32634327 10.1161/CIRCEP.119.008253

[38] Leenhardt A, Lucet V, Denjoy I et al (1995) Catecholaminergic polymorphic ventricular tachycardia in children. A 7-year follow-up of 21 patients. Circulation 91:1512–1519. 10.1161/01.cir.91.5.1512 7867192 10.1161/01.cir.91.5.1512

