# Supplementary material for: Epidemiology of paediatric inherited arrhythmogenic diseases under “Real World” conditions: findings from a 10-year longitudinal study in Eastern Austria
Source: Eur J Pediatr. 2025 Dec 3;184(12):815. doi: 10.1007/s00431-025-06646-z (PMC12672594; doi:10.1007/s00431-025-06646-z)
Supplement: Supplementary file 1 — (DOCX 60.7 KB) [file 431_2025_6646_MOESM1_ESM.docx]

| LQTS | | | | | | | | | | |
| --- | --- | --- | --- | --- | --- | --- | --- | --- | --- | --- |
| LQT 1 | Gender | Age at diagnosis | Age at reference date | Path to diagnosis | Event/Family history | ECG | Mutation | Nucleotide Change | Amino Acid Change | Schwartz-Score |
| N = 27 | m | 5 | 9 | Event | syncope, exercise induced | QTc 506 ms | KCNQ1 | c.582C>T | p.p.Arg195Trp | 6 |
|  | m | 8 | 13 | family history |  | QTc 480 ms | KCNQ1 | c.1893dupC | p.Arg632Glnfs*20 | 4 |
|  | f | 5 | 13 | family history |  | QTc 503 ms | KCNQ1 | n.a. |  | 4 |
|  | f | 3 | 12 | family history |  | QTc 522 ms | KCNQ1 | c.535G>A | p.Gly179Ser | 4 |
|  | f | 10 | 11 | family history |  | QTc 504 ms | KCNQ1 | n.a. |  | 4 |
|  | f | 6 | 6 | family history |  | QTc 480 ms | KCNQ1 | c.1032G>A | p.Ala344= | 4 |
|  | f | 1 | 4 | family history |  | QTc 481 ms | KCNQ1 | c.1032G>A | p.Ala344= | 4 |
|  | m | 12 | 12 | family history | mother ACA | QTc 462 ms | KCNQ1 | c.4771G>A | p.Glu1591Lys | 3 |
|  | f | 14 | 18 | incidental ECG |  | QTc 513 ms | KCNQ1 | c.1032G>A | p.Ala344= | 4 |
|  | f | 16 | 18 | incidental ECG |  | QTc 486 ms | KCNQ1 | c.1486_1487delCT | p.Leu496Alafs*19 | 4 |
|  | f | 12 | 16 | incidental ECG |  | QTc 471 ms | KCNQ1 | c.1032G>A | p.Ala344= | 4 |
|  | m | 12 | 16 | incidental ECG |  | QTc 506 ms | KCNQ1 | c.604G>A | p.Gly202Ser | 3 |
|  | f | 5 | 15 | incidental ECG |  | QTc 509 ms | KCNQ1 | c.1553G>A | p.Arg518His | 4 |
|  | f | 9 | 14 | incidental ECG |  | QTc 526 ms | KCNQ1 | c.1553G>A | p.Arg518His | 4 |
|  | f | 4 | 14 | incidental ECG |  | QTc 534 ms | KCNQ1 | c.845T>C | p.Leu282Pro | 5 |
|  | m | 10 | 13 | incidental ECG |  | QTc 516 ms | KCNQ1 | c.514G>A | p.Val172Met | 4 |
|  | f | 4 | 13 | incidental ECG |  | QTc 490 ms | KCNQ1 | c.973G>A | p.Gly325Arg | 5 |
|  | m | 8 | 12 | incidental ECG |  | QTc 517 ms | KCNQ1 | c.1031C>T | p.Ala344Val | 3 |
|  | m | 8 | 12 | incidental ECG |  | QTc 464 ms | KCNQ1 | c.604G>A | p.Asp202Asn | 2 |
|  | f | 2 | 9 | incidental ECG |  | QTc 483 ms | KCNQ1 | microchromosomal deletion del11p15.5 | -/- | 3 |
|  | m | 5 | 8 | incidental ECG |  | QTc 484 ms | KCNQ1 | c.1486_1487delCT | p.Leu496Alafs*19 | 3 |
|  | f | 1 | 7 | incidental ECG |  | QTc 522 ms | KCNQ1 | c.520C>T | p.Arg174Cys | 3,5 |
|  | f | 2 | 7 | incidental ECG |  | QTc 465 ms | KCNQ1 | c.604G>A | p.Asp202Asn | 4 |
|  | m | 4 | 7 | incidental ECG |  | QTc 472 ms | KCNQ1 | c.1772G>T | p.Arg591Leu | 3 |
|  | m | 1 | 5 | incidental ECG |  | QTc 512 ms | KCNQ1 | c.535G>A | p.Gly179Ser | 4 |
|  | f | 0 | 3 | incidental ECG |  | QTc 505 ms | KCNQ1 + MYH7 | c.1096C>T | p.Arg366Cys | 3 |
|  | f | 2 | 3 | incidental ECG |  | QTc 468 ms | KCNQ1 | c.1772G>T | p.Arg591Leu | 3 |
|  | | | | | | | | |  |  |
| LQT 2 | Gender | Age at diagnosis | Age at reference date | Path to diagnosis | Event/Family history | ECG | Mutation | Nuclotide Change | Amino Acid Change | Schwartz-Score |
| N = 16 | m | 11 | 18 | Event | syncope, stress induced | QTc 483 ms | KCNH2 | c.1750G>A | p.Gly584Ser | 5 |
|  | m | 12 | 14 | family history |  | QTc 462 ms | KCNH2 | c.1750G>A | p.Gly584Ser | 5 |
|  | m | 12 | 13 | family history |  | QTc 498 ms | KCNH2 | c.1817C>T | p.Ser606Phe | 4 |
|  | m | 6 | 8 | family history |  | QTc 496 ms | KCNH2 | c.2775dupG | p.Pro926Alafs*14 | 4 |
|  | m | 0 | 5 | family history | grandmother SCD | QTc 483 ms | KCNH2 | dup exon12(400bp) | -/- | 4 |
|  | f | 15 | 16 | family history |  | QTc 467 ms | KCNH2 | c.526C>T | p.Arg176Trp | 5 |
|  | f | 14 | 15 | family history |  | QTc 465 ms | KCNH2 | c.1817C>T | p.Ser606Phe | 5 |
|  | m | 0,3 | 8 | family history | grandmother SCD | QTc 513 ms | KCNH2 | dup exon12(400bp) | -/- | 4 |
|  | m | 0,5 | 3 | family history | grandmother SCD | QTc 471 ms | KCNH2 | dup exon12(400bp) | -/- | 3 |
|  | m | 0,5 | 18 | incidental ECG |  | QTc 506 ms | KCNH2 | c.1899C>A | p.Asn633Lys | 3 |
|  | f | 12 | 17 | incidental ECG |  | QTc 482 ms | KCNH2 | c.2350C>T | p.Arg784Trp | 5 |
|  | f | 11 | 13 | incidental ECG |  | QTc 473 ms | KCNH2 | c.526C>T | p.Arg176Trp | 3 |
|  | f | 2 | 12 | incidental ECG |  | QTc 478 ms | KCNH2 | c.1919_1921delTCT | p.Ser640del | 2 |
|  | m | 7 | 12 | incidental ECG |  | QTc 468 ms | KCNH2 | c.2350C>T | p.Arg784Trp | 2 |
|  | m | 8 | 10 | incidental ECG |  | QTc 483 ms | KCNH2 | c.2775dupG | p.Pro926Alafs*14 | 5 |
|  | m | 3 | 6 | incidental ECG |  | QTc 470 ms | KCNH2 | c.1817C>T | p.Ser606Phe | 2 |
|  | | | | | | | | |  |  |
| LQT 3-8 | Gender | Age at diagnosis | Age at reference date | Path to diagnosis | Event/Family history | ECG | Mutation | Nucleotide Change | Amino Acid Change | Schwartz-Score |
| N = 9 | m | 8 | 10 | Event | ACA, swimming | QTc 509 ms | CACNA1C | c.4075G>C | p.Ala1359Pro | 6 |
|  | f | 5 | 5 | Event | syncope | QTc 560 ms | CACNA1C | c.3487G>A | p.Gly1163Ser | 4 |
|  | f | 13 | 16 | Event | syncope | QTc 464 ms | KCNJ2 | c.652C>T | p.Arg218Trp | 3 |
|  | f | 0 | 11 | family history | ALTE | QTc 468 ms | ANK2 | n.a | n.a | 2 |
|  | f | 9 | 10 | incidental ECG | Polymorphic VTs | QTc 460 ms | pending |  |  |  |
|  | f | 6 | 9 | incidental ECG | Polymorphic VTs, mother ICD | QTc 467 ms | KCNJ2 | c.652C>T | p.Arg218Trp | 1 |
|  | f | 1 | 2 | incidental ECG | muscular weakness | QTc 460 ms | CACNA1C | c.1861T>C | pCys621Arg | 1 |
|  | m | 1 | 4 | incidental ECG |  | QTc 559 ms | CACNA1C, EMC1 | c.245C>T  c.560G>A  c.6667A>G | p.Thr2223Ala | 4,5 |
|  | m | 10 | 18 | incidental ECG |  | QTc 490 ms | SCN5A | c.1943C>T | p.Pro648Leu | 1 |
|  | | | | | | | | |  |  |
| LQT phenotypical | Gender | Age at diagnosis | Age at reference date | Path to diagnosis | Event/Family history | ECG | Mutation | Nucleotide Change | Amino Acid Change | Schwartz-Score |
| N = 3 | f | 13 | 17 | incidental ECG |  | QTc 511 ms | negative |  |  | 4 |
|  | f | 9 | 13 | incidental ECG |  | QTc 523 ms | negative |  |  | 3 |
|  | f | 13 | 13 | incidental ECG |  | QTc 553 ms | negative |  |  | 3,5 |
|  | | | | | | | | |  |  |
| LQT VUS | Gender | Age at diagnosis | Age at reference date | Path to diagnosis | Event/Family history | ECG | Mutation | Nucleotide Change | Amino Acid Change | Schwartz-Score |
| N = 8 | m | 6 | 9 | Event | syncope, exercise induced | QTc 506 ms | KCNQ1 | c.508G>A | p.Gly170Ser | 5 |
|  | m | 8 | 12 | family history |  | QTc 508 ms | KCNQ1 | c.1699G>C | p.Gly567Arg | 4 |
|  | m | 10 | 18 | incidental ECG |  | QTc 512 ms | KCNH2 | c.1899C>A | p.Asn633Lys | 3 |
|  | m | 9 | 15 | incidental ECG |  | QTc 465 ms | SCN5A | c.1180C>T | p.Arg394Cys | 2 |
|  | f | 1 | 7 | incidental ECG |  | QTc 522 ms | KCNQ1 | c.674C>T | p.Thr225Met | 3 |
|  | f | 14 | 18 | incidental ECG |  | QTc 482 ms | KCNJ5 | c.259C>T | p.Leu87Phe | 3 |
|  | m | 3 | 18 | incidental ECG |  | QTc 500 ms | KCNH2 | c.1899C>A | p.Asn633Lys | 3 |
|  | m | 9 | 13 | incidental ECG |  | QTc 466 ms | KCNQ1 | c.508G>A | p.Gly170Ser | 2 |

Supplementary Table 1a: Clinical characteristics and results of genetic analysis of LQTS patients

| BrS | | | | | | | | | | | | | | | | |
| --- | --- | --- | --- | --- | --- | --- | --- | --- | --- | --- | --- | --- | --- | --- | --- | --- |
| BrS | Gender | | Age at diagnosis | | Age at reference date | | Path to diagnosis | | Event, family history | | ECG | Mutation | | | Nucleotide Change | Amino Acid Change |
| N = 33 | m | | 9 | | 15 | | Event | | syncope (fever) | | normal | SCN5A  CACNA1C | | | c.998+5G>A  c.911T>C | -/-  p.Ile304Thr |
|  | m | | 17 | | 17 | | Event | | Syncope exercise | | BrS-like ECG | negative | | |  |  |
|  | f | | 4 | | 11 | | family history | | mother ICD | | BrS Type I ECG (fever) | SCN5A | | | c.718G>A | p.Val240Met |
|  | m | | 3 | | 16 | | family history | | mother BrS + ICD | | BrS Type I ECG | SCN5A | | | c.2437-1G>T | -/- |
|  | f | | 2 | | 4 | | family history | |  | | BrS Type I ECG | SCN5A | | | c.4912C>T | p.Arg1638Cys |
|  | m | | 12 | | 19 | | family history | | father BrS + ICD | | BrS-like ECG | AKAP9 | | | c.5660A>T | p.Gln1887Leu |
|  | m | | 13 | | 17 | | family history | | father BrS + ICD | | BrS-like ECG | AKAP9 | | | c.5660A>T | p.Gln1887Leu |
|  | m | | 13 | | 18 | | family history | |  | | BrS-like ECG | SCN5A | | | c.1066G>A | p.Asp356Asn |
|  | f | | 13 | | 17 | | family history | |  | | BrS-like ECG | SCN5A | | | c.4909C>T | p.Arg1637Ter |
|  | f | | 9 | | 16 | | family history | | mother BrS + ICD | | BrS-like ECG | SCN5A | | | c.718G>A | p.Val240Met |
|  | m | | 11 | | 13 | | family history | | grandmother SCD | | BrS-like ECG | SCN5A | | | c.4981G>A | p.Gly1661Arg |
|  | m | | 0 | | 3 | | family history | | grandfather SCD | | BrS-like ECG | SCN5A | | | c.5329G>A | p.Val177Met |
|  | m | | 4 | | 10 | | family history | | father ACA | | BrS-like ECG | SCN5A | | | c.311G>A | p.Arg104Gln |
|  | f | | 3 | | 9 | | family history | | uncle SCD | | BrS-like ECG | SCN5A | | | n.a |  |
|  | m | | 7 | | 10 | | family history | | father ICD + ajmaline +++ | | BrS-like ECG | index patient negative | | |  |  |
|  | m | | 1 | | 5 | | family history | |  | | BrS-like ECG | SCN5A | | | c.311G>A | p.Arg104Gln |
|  | f | | 6 | | 16 | | family history | |  | | normal | SCN5A | | | c.4845C>G | p.Tyr1615Term |
|  | f | | 16 | | 19 | | family history | | father SCD | | normal | SCN5A | | | c.2047T>G | p.Cys683Gly |
|  | f | | 7 | | 12 | | family history | | uncle SCD | | normal | SCN5A | | | n.a |  |
|  | f | | 4 | | 11 | | family history | |  | | normal | SCN5A | | | c.311C>A | p.Arg104Gln |
|  | m | | 1 | | 7 | | family history | |  | | normal | SCN5A | | | c.311C>A | p.Arg104Gln |
|  | f | | 2 | | 5 | | family history | | grandfather ICD, father ajmaline +++ | | normal | negative | | |  |  |
|  | f | | 6 | | 8 | | family history | | father ICD + ajmaline +++ | | normal | index patient negative | | |  |  |
|  | m | | 2 | | 4 | | family history | | father ICD + ajmaline +++ | | normal | index patient negative | | |  |  |
|  | f | | 1 | | 4 | | family history | |  | | normal | negative | | |  |  |
|  | m | | 0 | | 3 | | family history | | father ICD + ajmaline +++ | | normal | negative | | |  |  |
|  | f | | 15 | | 18 | | family history | | father ICD | | normal | genetic analysis refused | | |  |  |
|  | m | | 10 | | 16 | | family history | | father ajmaline +++ | | normal | genetic analysis refused | | |  |  |
|  | m | | 1 | | 5 | | incidental ECG | |  | | BrS-like ECG | genetic analysis refused | | |  |  |
|  | m | | 12 | | 16 | | incidental ECG | |  | | BrS-like ECG | SCN5A | | | c.4981G>A | p.Gly1661Arg |
|  | m | | 8 | | 8 | | incidental ECG | |  | | BrS-like ECG | pending | | |  |  |
|  | m | | 3 | | 4 | | incidental ECG | |  | | BrS Typ I ECG (fever) | negative | | |  |  |
|  | m | | 9 | | 13 | | incidental ECG | |  | | BrS Typ I ECG (fever) | negative | | |  |  |
|  | | | | | | | | | | | | | | | |  |
| BrS VUS | Gender | Age at diagnosis | | Age at reference date | | Path to diagnosis | | Event/Family history | | ECG | | | Mutation | Nucleotide Change | | Amino Acid Change |
|  | f | 10 | | 16 | | family history | |  | | normal | | | SCN5A | c.4847T>A | | p.Leu1616Gln |
|  | m | 9 | | 15 | | incidental ECG | |  | | QTc 465 ms | | | SCN5A | c.1180C>T | | p.Arg394Cys |

Supplementary Table 1b: Clinical characteristics and results of genetic analysis of BrS patients

| ARVC | | | | | | | | | | |
| --- | --- | --- | --- | --- | --- | --- | --- | --- | --- | --- |
| ARVC | Gender | Age at diagnosis | Age at reference date | Path to diagnosis | Event/Family history | ECG | Mutation | Nucleotide Change | Amino Acid Change |  |
| N = 17 | m | 12 | 15 | Event | syncope | PVCs, QRS fragmentation | negative |  |  |  |
|  | f | 13 | 15 | Event | syncope stress-induced | PVCs | PKP2 | c.1951C>T | p.Arg651Ter |  |
|  | m | 10 | 13 | Event | syncope | PVCs | RYR2 | c.877C>T | p.Arg293Cys |  |
|  | f | 10 | 15 | family history |  | normal | DSG2 | c.3059_3062delAGAG | p.Glu1020Alafs*18 |  |
|  | m | 8 | 13 | family history |  | PVCs | DSG2 | c.3059_3062delAGAG | p.Glu1020Alafs*18 |  |
|  | m | 10 | 13 | family history | brother ACA | normal | DSG2 | c.938_941delins59 | p.Ala313GlufsTer10 |  |
|  | f | 14 | 17 | family history | brother SCD | nsVTs, QRS fragmentation, low voltage | DSG2 | c.938_941delins59 | p.Ala313GlufsTer10 |  |
|  | f | 6 | 9 | family history | mother ACA | normal | DSP | c.5830C>T | p.Gln1944Ter |  |
|  | f | 17 | 18 | family history | father SCD | PVCs, QRS fragmentation | DSP | n.a. |  |  |
|  | m | 12 | 14 | family history |  | normal, SCD familial | DSP | c.939+1G>A | -/- |  |
|  | m | 11 | 14 | family history | mother ACA | normal | DSP | c.5830C>T | p.Gln1944Ter |  |
|  | f | 11 | 18 | family history | brother ACA | normal | PKP2 | c.1378+1G>C | -/- |  |
|  | f | 1 | 1 | family history |  | normal | PKP2 | c.2146-1G>C | -/- |  |
|  | m | 0 | 11 | family history | father + uncle SCD | normal | TMEM43 | c.1073C>T | p.Ser358Leu |  |
|  | f | 9 | 14 | incidental ECG |  | PVCs | negative |  |  |  |
|  | f | 0,3 | 9 | incidental ECG |  | QTc 460 ms | PKP2 | c.184C>A | p.Gln62Lys |  |
|  | | | | | | | | |  |  |
| ARVC VUS | Gender | Age at diagnosis | Age at reference date | Path to diagnosis | Event/Family history | ECG | Mutation | Nucleotide Change | Amino Acid Change |  |
| N = 2 | f | 1 | 9 | incidental ECG |  | QTc 469 ms | AKAP9 | c.8403T>G | p.Ile2801Met |  |
|  | m | 15 | 18 | family history | brother SCD | epsilon wave | PKP2 | c.1693A>G | p.Met565Val |  |

Supplementary Table 1c: Clinical characteristics and results of genetic analysis of ARVC patients

| CPVT, Laminopathy and others | | | | | | | | |  |
| --- | --- | --- | --- | --- | --- | --- | --- | --- | --- |
| CPVT | Gender | Age at diagnosis | Age at reference date | Path to diagnosis | Event, family history | ECG | Mutation | Nucleotide change | Amino Acid Change |
|  | m | 10 | 16 | Event | syncope, exercise-induced | PVCs | CASQ2 | c.733C>T | p.Gln245Ter |
|  | m | 7 | 12 | Event | syncope, exercise-induced | PVCs | CASQ2 | c.733C>T | p.Gln245Ter |
|  | | | | | | | |  |  |
| CPVT VUS | Gender | Age at diagnosis | Age at reference date | Path to diagnosis | Event, family history | ECG | Mutation | Nucleotide Changes | Amino Acid Change |
|  | m | 8 | 12 | family history |  | QTc 508 ms | RYR2 | c.877C>T | p.Arg293Cys |
|  | | | | | | | | |  |
| Lamin A/C | f | 12 | 18 | family history | PVCs, low voltage | PVCs, Low voltage | LMNA | c.1130G>A | p.Gly377Asp |
| Short QTS | m | 7 | 12 | Event | syncope, exercise-induced | PVCs | CASQ2 | c.733C>T |  |
|  | m | 14 | 16 | Event | syncope, nsVTs, aFib | QTc 331 ms | negative |  |  |
| V - Fib | m | 15 | 18 | Event | ACA, V-Fib | V-Fib | SCN5A | c.4525C>T | p.Pro1509Ser |
| Sinus node disease | m | 12 | 18 | family history | sinus bradycardia | sinus bradycardia | KCNJ5 | c.303G>C | p.Glu101Asp |

Supplementary Table 1d: Clinical characteristics and results of genetic analysis of CPVT, Lamin A/C and other patients

Supplementary Table 1a - d

Clinical characteristics and results of genetic analysis of all IADs patients.

(IADs Inherited arrhythmogenic diseases, LQTS Long QT syndrome, BrS Brugada syndrome, ARVC arrhythmogenic right ventricular cardiomyopathy, CPVT catecholaminergic polymorphic ventricular tachycardia, nsVTs non sustained ventricular tachycardia, Lamin A/C Laminopathy A/C, SQTS short QT syndrome, V-Fib ventricular fibrillation, PVCs premature ventricular complexes, ALTE apparent life-threatening event, ACA aborted cardiac arrest).
